# Invasive Cancer Incidence — United States, 2010

**Published:** 2014-03-28

**Authors:** S. Jane Henley, Simple Singh, Jessica King, Reda Wilson, Blythe Ryerson

**Affiliations:** 1 Division of Cancer Prevention and Control, National Center for Chronic Disease Prevention and Health Promotion, CDC

Cancer has many causes, some of which can, at least in part, be avoided through interventions known to reduce cancer risk (1). *Healthy People 2020* objectives call for reducing colorectal cancer incidence to 38.6 per 100,000 persons, reducing late-stage breast cancer incidence to 41.0 per 100,000 women, and reducing cervical cancer incidence to 7.1 per 100,000 women (2). To assess progress toward reaching these *Healthy People 2020* targets, CDC analyzed data from U.S. Cancer Statistics (USCS) for 2010. USCS includes incidence data from CDC’s National Program of Cancer Registries and the National Cancer Institute’s Surveillance, Epidemiology, and End Results (SEER) program and mortality data from the National Vital Statistics System (3). In 2010, a total of 1,456,496 invasive cancers were reported to cancer registries in the United States (excluding Arkansas and Minnesota), an annual incidence rate of 446 cases per 100,000 persons, compared with 459 in 2009 (4). Cancer incidence rates were higher among men (503) than women (405), highest among blacks (455), and ranged by state from 380 to 511 per 100,000 persons. Many factors, including tobacco use, obesity, insufficient physical activity, and human papilloma virus (HPV) infection, contribute to the risk for developing cancer, and differences in cancer incidence indicate differences in the prevalence of these risk factors. These differences can be reduced through policy approaches such as the Affordable Care Act,* which could increase access for millions of persons to appropriate and timely cancer preventive services, including help with smoking cessation, cancer screening, and vaccination against HPV (5).

Invasive cancers include all cancers except in situ cancers (other than in the urinary bladder) and basal and squamous cell skin cancers. Data on new cases of invasive cancer diagnosed during 2010 were obtained from population-based cancer registries affiliated with the National Program of Cancer Registries and SEER programs in each state and the District of Columbia (DC) (3). Data from all states except Arkansas and Minnesota met USCS publication criteria for 2010†; consequently, data in this report cover 97% of the U.S. population. Cases were first classified by anatomic site using the *International Classification of Diseases for Oncology, Third Edition* (ICD-O-3). Cases with hematopoietic histologies were further classified using the *WHO Classification of Tumours of Haematopoietic and Lymphoid Tissues, Fourth Edition*. Breast cancers also were characterized by stage at diagnosis using SEER Summary Stage 2000§; late-stage cancers include those diagnosed at a regional or distant stage.

Population denominators for incidence rates are race-specific, ethnicity-specific, and sex-specific county population estimates from the 2010 U.S. Census, as modified by SEER and aggregated to the state and national level.¶ Annual incidence rates per 100,000 population were age-adjusted by the direct method to the 2000 U.S. standard population.

In 2010, a total of 1,456,496 invasive cancers were diagnosed and reported to central cancer registries in the United States (excluding Arkansas and Minnesota), including 745,383 among males and 711,113 among females (Table). The age-adjusted annual incidence for all cancers was 446 per 100,000 population; 503 per 100,000 in males (compared with 524 in 2009) and 405 per 100,000 in females (compared with 414 in 2009). Among persons aged ≤19 years, 14,276 cancer cases were diagnosed in 2010 (Table). By age group, rates per 100,000 population in 2010 were 17.5 among persons aged ≤19 years, 152.3 among those aged 20–49 years, 804.8 among those aged 50–64 years, 1,816.2 among those aged 65–74 years, and 2,209.9 among those aged ≥75 years (Table).

By cancer site, rates were highest for cancers of the prostate (126.1 per 100,000 men), female breast (118.7 per 100,000 women), lung and bronchus (61.7 per 100,000 persons), and colon and rectum (40.4 per 100,000 persons) (Table). These four sites accounted for half of cancers diagnosed in 2010, including 196,038 prostate cancers, 206,966 female breast cancers, 201,144 lung and bronchus cancers, and 131,607 colon and rectum cancers. In 2010, the cervical cancer incidence rate was 7.5 per 100,000 women, representing 11,818 reported cancers.

In 2010, the top 10 cancer sites differed by sex and racial/ethnic group (Figure 1). Among men, prostate, lung, and colorectal cancers were the first, second, and third most common cancers in all racial/ethnic groups. Among women, breast cancer was the most common cancer among all racial/ethnic groups, followed by lung, colorectal, and uterine cancers in all racial/ethnic groups, except among Hispanic women, among whom colorectal cancer was more common than lung cancer, and Asian/Pacific Islander women, among whom the most common cancers were colorectal, lung, and thyroid (Figure 1). At 49.8 per 100,000 women, the incidence of late-stage breast cancer was highest among black women, compared with 22.8 for American Indian/Alaska Native women, 28.6 for Asian/Pacific Islander women, 33.6 for Hispanic women, and 40.9 for white women.

By state in 2010, all-sites cancer incidence rates ranged from 380.4 to 510.7 per 100,000 persons (Figure 2). State site-specific cancer incidence rates ranged from 90.6 to 187.0 per 100,000 men for prostate cancer, 106.3 to 142.9 per 100,000 women for female breast cancer, 26.8 to 97.3 per 100,000 persons for lung cancer, 31.5 to 51.3 per 100,000 persons for colorectal cancer, and 5.0 to 11.2 per 100,000 women for cervical cancer (Figure 2). *Healthy People 2020* targets were reached in 15 states (compared with seven in 2009) for incidence of colorectal cancer and in 24 states (compared with 19 in 2009) for incidence of cervical cancer.

## Discussion

This report provides estimates of cancer incidence for 2010 in the United States and shows that *Healthy People 2020* targets were reached in 15 states for reduced colorectal cancer incidence and 24 states for reduced cervical cancer incidence. For the first time, lung cancer was the second most common cancer among Hispanic men, surpassing colorectal cancer, although it is too soon to determine whether this trend is likely to continue. Fewer cancers were reported to cancer registries in 2010 than in 2009 (4). Decreases in case counts might reflect actual changes in cancer incidence, changes in the detection of cancer resulting from variations in delivery or use of cancer screening tests, recent decreases in health care use (6) because some cancers are diagnosed incidentally, or a drop in the completeness of case ascertainment at the registry level. Ascertaining the specific reason is difficult, and CDC and the National Cancer Institute continue to monitor these trends.

Policy approaches can enhance evidence-based interventions to reach *Healthy People 2020* targets (1,5). For example, most cervical cancers could be prevented through HPV vaccination and effective screening (7). However, only 33% of girls aged 13–17 years received the recommended 3-dose HPV vaccine series in 2012; by increasing this to 80%, an estimated 53,000 cases of cervical cancer could be prevented over the lifetimes of girls aged ≤12 years.** In 2010, 83% of women received recommended cervical cancer screening.†† Section 1001 of the Affordable Care Act removes the financial barriers to these and other preventive services by requiring nonexempted private health insurance plans to cover, with no deductibles or copayments, a collection of clinical preventive services. Those services include vaccinations recommended by the Advisory Committee on Immunization Practices and A-rated or B-rated clinical preventive services recommended by the U.S. Preventive Services Task Force, such as cancer screening and tobacco cessation counseling.§§ Administrative rules promulgated by the U.S. Department of Health and Human Services established requirements for similar preventive services coverage for enrollees in expanded state Medicaid plans.¶¶

What is already known on this topic?

In the United States in 2009, the incidence rate of invasive cancer was 524 per 100,000 among men and 414 among women. By state, all-sites cancer incidence rates ranged from 387 to 509 per 100,000 population. *Healthy People 2020* targets were reached in seven states for reduced incidence of colorectal cancer and in 19 states for reduced incidence of cervical cancer.

What is added by this report?

National cancer surveillance data indicate that 1,456,496 new cases of invasive cancer were diagnosed in the United States (excluding Arkansas and Minnesota) in 2010, an annual incidence rate of 503 cases per 100,000 among men and 405 among women, both lower than in 2009. As in 2009, cancer incidence rates were highest (455 per 100,000 persons) among black persons, largely reflecting higher rates of cancers of the prostate and female breast. By state, all-sites cancer incidence rates ranged from 380 to 511 per 100,000 population. *Healthy People 2020* targets were reached in 15 states for reduced incidence of colorectal cancer and in 24 states for reduced incidence of cervical cancer.

What are the implications for public health practice?

Differences in cancer incidence reflect differences in the prevalence of cancer risk factors. Evidence-based interventions to reduce these differences can be enhanced through policy approaches such as the Affordable Care Act of 2010, which could increase access for millions of persons to appropriate and timely cancer preventive services such as help with smoking cessation, cancer screening, and vaccination against the human papillomavirus.

CDC annually provides cancer surveillance data via several data release products, including USCS, CDC WONDER, State Cancer Profiles, and data from the National Center for Health Statistics (NCHS) Research Data Centers.*** These data can be useful in several ways.††† First, these data can guide the planning and evaluation of cancer prevention and control programs. The DC Cancer Registry, for example, found that the rate of colorectal cancer incidence was highest among residents in wards 7 and 8. In response, the DC Cancer Consortium and the DC Comprehensive Cancer Control Program funded a citywide program, focusing on those two wards, to provide free colorectal cancer screening tests to persons without health insurance. Second, these data can assist long-term planning for cancer diagnostic and treatment services. For example, a linkage of 13 cancer registries with the Scientific Registry of Transplant Recipients showed that organ transplant patients have a higher risk for cancer than the general population and might benefit from rigorous cancer screening during follow-up (8). Third, these data can help public health officials set priorities for allocating health resources. In Kentucky, for example, cancer registry data showed high and increasing rates of colorectal cancer incidence. In response, state and regional cancer control representatives aggressively promoted colorectal cancer screening; subsequently, screening rates increased from 35% in 1999 to 64% in 2008, and incidence rates decreased from 69 per 100,000 persons in 2001 to 56 in 2009 (9).

The findings in this report are subject to at least two limitations. First, analyses based on race and ethnicity might be biased if race and ethnicity were misclassified; ongoing efforts are made to ensure that this information is as accurate as possible.§§§ Second, delays in cancer reporting might result in an underestimate of certain cancers; reporting delays are more common for cancers such as melanoma that are diagnosed and treated in nonhospital settings such as physicians’ offices (10).

National cancer surveillance data help public health officials monitor the cancer burden in the United States, identify populations with high cancer rates that might benefit most from targeted cancer prevention efforts, and track progress toward the national cancer objectives set forth in *Healthy People 2020*.

## Figures and Tables

**FIGURE 1:**
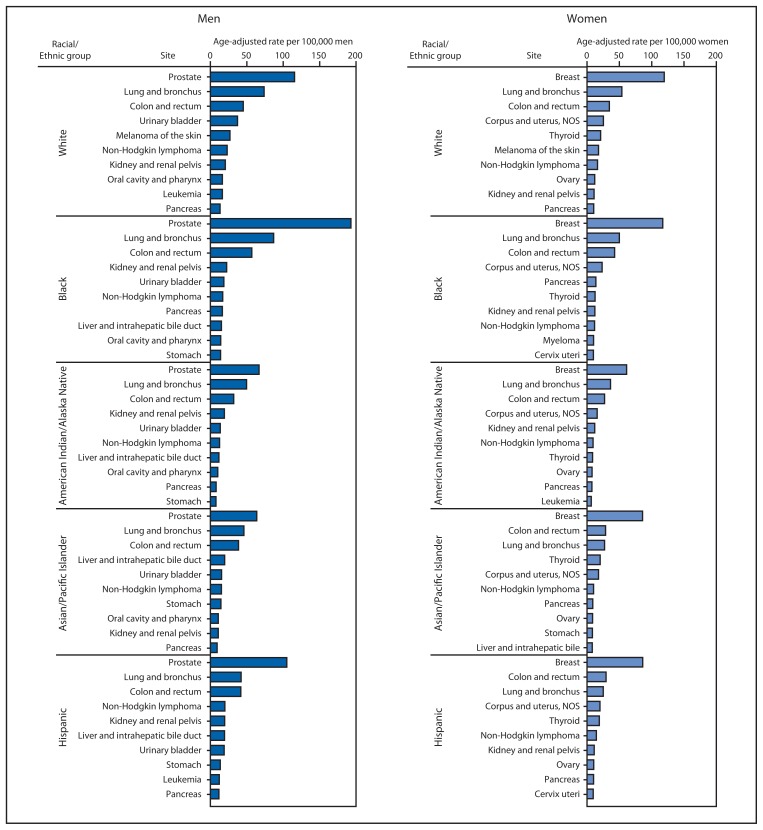
Rate^*^ of invasive cancer for 10 primary sites with the highest rates within racial/ethnic groups,^†^ by sex — National Program of Cancer Registries and Surveillance, Epidemiology, and End Results program, United States, 2010^§^ **Abbreviation:** NOS = not otherwise specified. ^*^ Per 100,000 persons, age-adjusted to the 2000 U.S. standard population. ^†^ Racial categories are not mutually exclusive from Hispanic ethnicity. Rates are not presented for cases with unknown or other race. ^§^ Compiled from cancer registries that meet the data quality criteria for all invasive cancer sites combined (covering approximately 97% of the U.S. population). Excludes basal and squamous cell carcinomas of the skin except when these occur on the skin of the genital organs, and in situ cancers other than urinary bladder.

**FIGURE 2:**
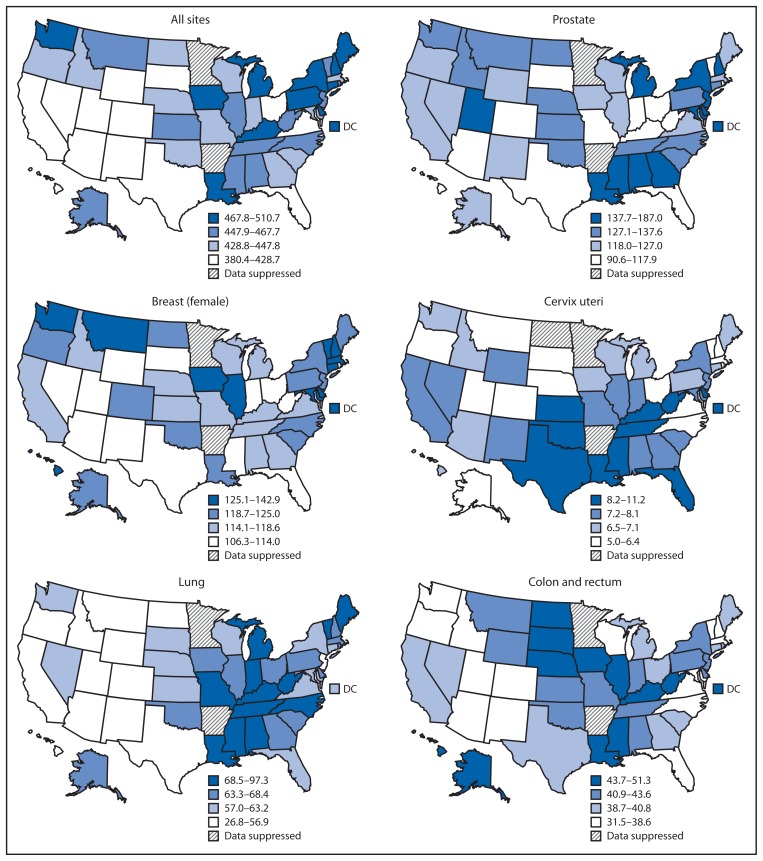
Rate^*^ of invasive cancer, by primary cancer site — National Program of Cancer Registries and Surveillance, Epidemiology, and End Results program, United States, 2010 ^*^ Per 100,000 persons, age-adjusted to the 2000 U.S. standard population.

**TABLE:** Number of invasive cancers* and annual rate,† by sex, primary site, racial/ethnic group,§ and age group — National Program of Cancer Registries, and Surveillance, Epidemiology, and End Results program, United States,¶ 2010

Characteristic	Overall			Men			Women		
		
	Rate	No.	(%)	Rate	No.	(%)	Rate	No.	(%)
**All cancers**	**445.5**	**1,456,496**		**502.7**	**745,383**		**405.1**	**711,113**	
Prostate	**NA**	**196,038**	**(13)**	126.1	196,038	(26)	NA	NA	
Female breast	**NA**	**206,966**	**(14)**	NA	NA		118.7	206,966	(29)
Late-stage female breast	**NA**	**71,691**		NA	NA		41.7	71,691	
Lung and bronchus	**61.7**	**201,144**	**(14)**	74.1	107,164	(14)	52.4	93,980	(13)
Colon and rectum	**40.4**	**131,607**	**(9)**	46.4	67,700	(9)	35.4	63,907	(9)
Cervix uteri	**NA**	**11,818**	**(1)**	NA	NA		7.5	11,818	(2)
**Racial/Ethnic group**									
White	**444.9**	**1,224,067**	**(84)**	495.2	625,371	(84)	409.9	598,696	(84)
Black	**454.6**	**157,085**	**(11)**	553.2	80,638	(11)	388.8	76,447	(11)
American Indian/Alaska Native	**270.3**	**7,361**	**(1)**	299.2	3,588	(<1)	251.9	3,773	(1)
Asian/Pacific Islander	**289.2**	**41,541**	**(3)**	307.6	18,730	(3)	279.7	22,811	(3)
Hispanic	**343.9**	**103,050**	**(7)**	390.4	50,281	(7)	314.9	52,769	(7)
**Age group (yrs)**									
≤19	**17.5**	**14,276**	**(1)**	18.6	7,739	(1)	16.4	6,537	(1)
20–49	**152.3**	**185,051**	**(13)**	113.5	69,184	(9)	190.6	115,867	(16)
50–64	**804.8**	**474,859**	**(33)**	874.3	251,046	(34)	740.6	223,813	(31)
65–74	**1,816.2**	**382,519**	**(26)**	2,234.1	218,334	(29)	1,455.0	164,185	(23)
≥75	**2,209.9**	**399,791**	**(27)**	2,802.4	199,080	(27)	1,823.1	200,711	(28)

**Abbreviation:** NA = not applicable.

* Excludes basal and squamous cell carcinomas of the skin except when these occur on the skin of the genital organs, and in situ cancers other than urinary bladder.

† Per 100,000 persons, age-adjusted to the 2000 U.S. standard population.

§ Racial categories are not mutually exclusive from Hispanic ethnicity. Rates are not presented for cases with unknown or other race.

¶ Compiled from cancer registries that meet the data quality criteria for all invasive cancer sites combined (covering approximately 97% of the U.S. population).

## Footnotes

* Patient Protection and Affordable Care Act of 2010. Pub. L. No. 114-48 (March 23, 2010), as amended through May 1, 2010. Available at http://www.healthcare.gov/law/full/index.html.

† Cancer registries demonstrated that cancer incidence data were of high quality by meeting the six USCS publication criteria: 1) case ascertainment is ≥90% complete, 2) ≤5% of cases are ascertained solely on the basis of a death certificate, 3) ≤3% of cases are missing information on sex, 4) ≤3% of cases are missing information on age, 5) ≤5% of cases are missing information on race, and 6) ≥97% of the registry’s records passed a set of single-field and inter-field computerized edits that test the validity and logic of data components. Additional information available at http://www.cdc.gov/uscs.

§ Additional information available at http://seer.cancer.gov/tools/ssm.

¶ Population estimates for 2010 incorporate bridged single-race estimates that are derived from the original multiple race categories in the 2010 Census. Additional information available at http://seer.cancer.gov/popdata/index.html and http://www.census.gov/popest/topics/methodology.

** Information available at http://www.cdc.gov/mmwr/preview/mmwrhtml/mm6234a1.htm.

†† Information available at http://www.cdc.gov/mmwr/preview/mmwrhtml/mm6103a1.htm.

§§ Information available at http://www.uspreventiveservicestaskforce.org/uspstf/uspsabrecs.htm.

¶¶ Medicaid and Children’s Health Insurance Programs: essential health benefits in alternative benefit plans, eligibility notices, fair hearing and appeal processes, and premiums and cost sharing; exchanges: eligibility and enrollment, 78 Fed. Reg. 42,160, 42,307 (July 15, 2013) (amending 42 C.F.R. ^§^440.360).

*** Additional information available at http://www.cdc.gov/cancer/npcr/datarelease.htm, http://wonder.cdc.gov, http://www.statecancerprofiles.cancer.gov/incidencerates/index.php, and http://www.cdc.gov/rdc/b1datatype/dt131.htm.

††† Additional information available at http://www.cdc.gov/cancer/npcr/value/index.htm.

§§§ Additional information available at http://www.cdc.gov/cancer/npcr/uscs/technical_notes/interpreting/race.htm.

## References

[1] Colditz GA, Wolin KY, Gehlert S (2012). Applying what we know to accelerate cancer prevention. Sci Transl Med.

[2] US Department of Health and Human Services (2011). Healthy people 2020.

[3] US Cancer Statistics Working Group (2013). United States cancer statistics: 1999–2010 incidence and mortality web-based report.

[4] CDC (2013). Invasive cancer incidence—United States, 2009. MMWR.

[5] Frieden TR (2013). Government’s role in protecting health and safety. N Engl J Med.

[6] Mortensen K, Chen J (2013). The Great Recession and racial and ethnic disparities in health services use. JAMA Int Med.

[7] Watson M, Saraiya M, Benard V (2008). Burden of cervical cancer in the United States, 1998–2003. Cancer.

[8] Engels EA, Pfeiffer RM, Fraumeni JF (2011). Spectrum of cancer risk among US solid organ transplant recipients. JAMA.

[9] Kentucky Cancer Consortium (2012). A KCC snapshot of colon cancer.

[10] Clegg LX, Feuer EJ, Midthune DN (2002). Impact of reporting delay and reporting error on cancer incidence rates and trends. J Natl Cancer Inst.

